# Immunostimulatory nanoparticles delivering cytokines as a novel cancer nanoadjuvant to empower glioblastoma immunotherapy

**DOI:** 10.1007/s13346-023-01509-2

**Published:** 2023-12-31

**Authors:** Flávia Sousa, Henry Lee, Mauro Almeida, Amelie Bazzoni, Barbara Rothen-Rutishauser, Alke Petri-Fink

**Affiliations:** 1 Adolphe Merkle Institute, University of Fribourg, Fribourg, Switzerland; 2 National Center of Competence in Research Bio-Inspired Materials, University of Fribourg, Fribourg, Switzerland; 3 Chemistry Department, University of Fribourg, Chemin du Musée 9, 1700 Fribourg, Switzerland

**Keywords:** Immunotherapy, Nanoparticles, Glioblastoma, IL-12, Cancer nanoadjuvant, Immunostimulatory cytokines

## Abstract

Glioblastoma (GBM) stands as a highly aggressive and deadly malignant primary brain tumor with a median survival time of under 15 months upon disease diagnosis. While immunotherapies have shown promising results in solid cancers, brain cancers are still unresponsive to immunotherapy due to immunological dysfunction and the presence of a blood–brain barrier. Interleukin-12 (IL-12) emerges as a potent cytokine in fostering anti-tumor immunity by triggering interferon-gamma production in T and natural killer cells and changing macrophages to a tumoricidal phenotype. However, systemic administration of IL-12 toxicity in clinical trials often leads to significant toxicity, posing a critical hurdle. To overcome this major drawback, we have formulated a novel nanoadjuvant composed of immunostimulatory nanoparticles (ISN) loaded with IL-12 to decrease IL-12 toxicity and enhance the immune response by macrophages and GBM cancer cells. Our in vitro results reveal that ISN substantially increase the production of pro-inflammatory cytokines in GBM cancer cells (e.g. 2.6 × increase in IL-8 expression compared to free IL-12) and macrophages (e.g. 2 × increase in TNF-α expression and 6 × increase in IL-6 expression compared to the free IL-12). These findings suggest a potential modulation of the tumor microenvironment. Additionally, our study demonstrates the effective intracellular delivery of IL-12 by ISN, triggering alterations in the levels of pro-inflammatory cytokines at both transcriptional and protein expression levels. These results highlight the promise of the nanoadjuvant as a prospective platform for resharing the GBM microenvironment and empowering immunotherapy.

**Graphical Abstract:**

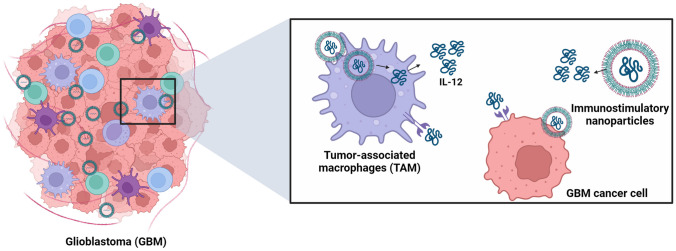

**Supplementary Information:**

The online version contains supplementary material available at 10.1007/s13346-023-01509-2.

## Introduction

Glioblastoma (GBM) is the most common and deadly primary malignant brain cancer in human adults. Its aggressive invasive growth, together with cellular and metabolic heterogeneity contribute to its resistance to therapy, making GBM an incurable disease with a poor prognosis [1]. Over the past decades, very little advances have been made to the GBM treatment plan. Standard procedure consists in surgery followed by radiotherapy and/or temozolomide plus adjuvant chemotherapy. Beyond the presence of the blood–brain barrier (BBB) that by itself hampers the delivery of therapeutics in the target site, several studies have confirmed that the extreme immunosuppressive microenvironment could be detrimental for the treatment success [2, 3]. The well-known immunosuppressive microenvironment makes immunotherapy in GBM a challenge predominantly due to the presence of significantly fewer differentiated T helper cells 0 (T_H_0), absence of natural killer (NK) and cytotoxic T cells, as well as M2 polarized cancer-associated macrophages (TAMs) [4].

Targeted delivery of immune-activating cytokines, such as IL-2 and IFN-α, have been approved by the Food and Drug Administration (FDA) for cancer immunotherapy in solid cancers since the 1980s [5]. Nevertheless, while immunotherapies have shown promising results in solid cancers, the success in treating GBM has been limited due to the immunosuppressive microenvironment and several mechanisms of physical constraints and molecular resistance. Previous efforts to deliver immune-activating cytokines as a strategy for GBM immunotherapy have failed due to the limitations in crossing the BBB, severe dose-limiting toxicity, and their uncontrollable regulation, being further hard to activate the migration of peripheral monocytes into the central nervous system (CNS) and inhibit the immunosuppressive microenvironment of GBM [6].

Interleukin 12 (IL-12) has been studied as one of the most potent cytokines for anti-cancer immunotherapy because it stimulates interferon (IFN)-γ production by T and natural killer (NK) cells, decreases the angiogenesis, and changes the cancer microenvironment from one that contains T_H_0 and M2-type phenotype macrophages to one richer in T_H_1 cells and inflammatory M1-type macrophages [7, 8]. Therefore, cytokines are regarded as potent immunoadjuvants capable of bolstering vaccine efficacy by fortifying the immune response elicited through vaccination [9]. In addition to that, previous studies have shown that intracavitary levels of IL-12 in GBM are lower than others anti-inflammatory cytokines (e.g., IL-8) resulting in the interest of using recombinant IL-12 as immunoadjuvant for GBM [10, 11]. Despite demonstrating enhanced survival in mice with the GL-261 mouse glioma model, systemic IL-12 trails were halted due to its significant toxicity, instability, and short half-life [7]. This outcome primarily resulted from IL-12’s poor tolerance as immune-activating cytokine, leading to cytokine release syndrome and compromised liver function upon intravenous administration [12, 13]. To harness the therapeutic benefits of IL-12 while addressing these challenges, innovative delivery systems capable of safeguarding IL-12 and prolonging its immunostimulatory impact could offer a promising solution to create a cancer nanoadjuvant.

Within delivery systems, poly lactic-co-glycolic (PLGA) nanoparticles (NPs) have gained significant attention for the delivery of small drugs, proteins, monoclonal antibodies, and nucleic acid [14]. PLGA is a biocompatible and biodegradable polymer approved by the Food and Drug Administration (FDA) and European Medicine Agency (EMA) for drug delivery systems, resulting in more than 15 FDA-approved Poly lactic acid (PLA)/PLGA-based drug systems currently available in the US market [15]. PLGA NPs have been used to deliver chemically unstable biomacromolecules, such as proteins and monoclonal antibodies, allowing their encapsulation and maintaining the biophysical structure and biological function [16].

In this study, the aim was to encapsulate IL-12 within PLGA and PLGA/1,2-dioleoyl-3-trimethylammonium-propan (DOTAP) nanoparticles. This approach was pursued to mitigate dose-limiting toxicity, enhance the short half-life, and facilitate a more effective expression of pro-inflammatory cytokines, surpassing the performance of free IL-12. To do so, immunostimulatory nanoparticles (ISN) and cationic polymeric nanoparticles (CPN) were formulated and composed of IL-12-loaded PLGA NPs and IL-12-loaded PLGA/DOTAP NPs, respectively. The study was specifically designed to achieve three specific objectives: (i) develop a cancer nanoadjuvant system for delivering the immunocytokine IL-12, (ii) address the dose-limiting toxicity associated with free IL-12, and (iii) enhance the immunostimulatory impact of IL-12 through an innovative delivery system. Our investigation enabled (i) prolonged interaction of PLGA with inflammatory and cancer cells and (ii) intracellular delivery of IL-12. The findings presented herein hold substantial promise in advancing GBM immunotherapy and pioneering a novel cancer nanoadjuvant.

## Materials and methods

### Preparation of immunostimulatory nanoparticles

ISN and empty PLGA NPs (PLGA NPs) were prepared through a modified solvent emulsification-evaporation method based on a previously reported w/o/w double emulsion technique [16, 17]. The first w/o emulsion was created with 20 mg of PLGA 50:50 (Resomer^®^ RG 504 from Sigma-Aldrich, Germany) dissolved in 1 mL of ethyl acetate (EA from Merck). The polymeric solution was homogenized with 100 µL of 2.5 µg/mL human IL-12 (Sigma-Aldrich, Germany) using a Vibra-Cell ultrasonic processor with 14% amplitude for 30s. Empty PLGA NPs were obtained with the same method, but 100 µL of water were added instead of human IL-12. The primary emulsion was added to 4 mL of 2% (w/w) Poly(vinyl alcohol) (PVA, Mowiol^®^ 4–88 31,000 Da from Sigma-Aldrich, Germany) and homogenized using the same conditions. Lastly, the second emulsion w/o/w was poured into 7.5 mL of 2% (w/w) PVA and left in the fume hood under magnetic stirring at 300rpm for 3h for EA evaporation. The purification of ISN and PLGA NPs was done immediately after formulation with three cycles of centrifugation. The centrifugation was performed at 20,000g for 30 min. After the third centrifugation, both formulations were redispersed in water and stored at 20mg/mL at 4 ºC.

### Preparation of cationic polymeric nanoparticles

Empty and loaded cationic polymeric nanoparticles (CPN) were prepared according to the previously described method with the same modifications. The organic phase was composed of a mixture of PLGA and DOTAP (Avanti® Polar Lipids). Three different concentrations between 0 and 15% (w/v) of DOTAP were added to the organic phase of the double emulsion. Twenty milligrams of PLGA/DOTAP were dissolved into 1 mL of chloroform due to low DOTAP solubility in EA. The remaining formulation protocol was kept the same and followed the previous protocol for ISN. The obtained CPN were washed three times with ultrapure water by centrifugation at 20,000g for 30 min.

### Characterization of nanoparticle formulations

ISN, PLGA NPs, empty CPN, and loaded CPN in suspension were characterized for their average particle size, polydispersity index (PDI), and zeta potential by dynamic light scattering (DLS) using an Anton Paar Litesizer 500 particle analyzer (Anton Paar, Graz, Austria). For the measurement, a set of three replicates with ten measurements were acquired at 90°, 22 C, and 532 nm. The samples were diluted with ultrapure water, NaCl 10 mM, phosphate-buffered solution (PBS) and Simulated Interstitial Fluid (SIF, Biochemazone, Canada) to an appropriate concentration.

The particle morphology was characterized by scanning electron microscopy (SEM) and transmission electron microscopy (TEM) using Tescan Mira 3 (Tescan, Brno – Kohoutovice, Czech Republic) and FEI Technai Spirit (FEI, Hillsboro, OR, USA) microscopes, respectively. All formulations were purified by centrifugation before SEM and TEM observation to eliminate residual surfactant (PVA). For SEM analysis, a small sample was placed on top of a glass slide mounted on the SEM stab with carbon tape. Before the observation, the SEM stab with the sample was vacuum-coated with a 2 mm layer of gold.

For TEM observations, a drop of 5uL of the sample was deposited onto the grid (200 mesh formvar carbon grid). The excess liquid was blotted after 1 min with filter paper. Immediately after, a 5 ul drop of the staining agent UranyLess EM Stain (EMS, Hatfield, PA 19440) was applied. The excess liquid was blotted after 1 min with filter paper. The grid was allowed to dry under room conditions 2 h before observations with the microscope.

### IL-12 encapsulation efficacy

The Encapsulation Efficacy (EE) of IL-12 was calculated by the indirect method after the first washing step of ISN and CPN. The free amount of IL-12 in the supernatant was quantified through a human IL-12 ELISA kit (R&D systems, UK). The supernatant of PLGA NPs and empty CPN were used as a control for the IL-12 quantification.

### Cell lines and culture media

Human glioblastoma (U87 MG) and murine macrophage cell line (J774A.1) were purchased from the American Type Cell Culture Collection (ATCC, USA). Human GBM cell line was cultured and maintained in Dulbecco’s Modified Eagle Medium (DMEM) supplemented with 10% fetal bovine serum (FBS) and 1% penicillin/streptomycin. Murine macrophage cell line was cultured and maintained in Roswell Park Memorial Institute 1640 (RPMI-1640, Gibco, Switzerland) supplemented with 10% GBS, 1% penicillin/streptomycin, and 1% glutamine. Both cell lines were kept at 37 ºC, 5% CO_2_, and 95% relative humidity and passaged twice per week between 80–90% confluency.

### In vitro cytotoxic assays

3-[4,5-dimethylthiazole-2-yl]-2,5-diphenyltetrazolium bromide (MTT) in vitro toxicology assay kit (Sigma-Aldrich, Germany) was used to measure the mitochondrial activity after exposure to all nanoformulations for U-87 MG and J774A.1 cells. For both cell lines, cytotoxicity caused by free IL-12, ISN, PLGA NPs, empty CPN, and loaded CPN was evaluated by collecting culture supernatant and determining L-lactate dehydrogenase (LDH) activity using an LDH kit (Roche, Germany). U-87 MG cells were seeded in 96-well plates at 2.5 × 10^5^ cells/mL, while J774A.1 cells were seeded at 1.5 × 10^5^ cells/mL for 24h in the respective cell culture media. After 24h of seeding, both cell lines were exposed to different nanoformulations at concentrations of 20 ng/mL of IL-12 for ISN and PLGA NPs and 5 ng/mL for empty CPN and loaded CPN. 0.2 vol. % Triton X-100 was added to the cell culture medium as a positive control [18]. In clinical trials, patients have received escalating doses of recombinant IL-12 from 30–700 ng/kg, while in vitro studies have shown promising results using IL-12 concentrations between 0.2 and 100 ng/mL[19–21]. In our pursuit to mitigate the off-target toxicity associated with IL-12, we selected concentrations of 5ng/mL and 20 ng/mL, falling within the therapeutic range. After 24h of incubation, the supernatant was used for LDH assay while cells were washed before being exposed to 0.5 mg/mL MTT solution. The MTT reaction was ended by the removal of the media and the addition of dimethyl sulfoxide. The final product’s absorbance was measured using a spectrophotometer (Benchmark Microplate reader, Biorad, Cressier, Switzerland) and following each kit’s protocol. All tests were performed with three biological replicates and 8 individual values.

### Analysis of cytokine expression

The secretion of immunostimulatory cytokines was studied in human glioblastoma cells and murine macrophages. Both cell lines were seeded in a six-well plate at a density of 2 × 10^5^ cells/mL. After 24h of seeding, cells were treated with 20 ng/mL of free IL-12, ISN, and PLGA NPs. PLGA NPs were incubated with the same dilution as ISN. LPS was used as a positive control at 10 ng/mL. After 24h of exposure, the concentration of released cytokines from both cell lines in the supernatants was measured by ELISA assays (R&D systems, UK) according to the manufacturer’s protocol. For the U-87 MG cell line, the concentration of extracellular IL-6, IL-8, VEGF, and IFN-γ was measured using human IL-6, human IL-8, VEGF, and IFN-γ DuoSet ELISA Kit. For the J774A.1 cell line, mouse IL-6, and TNF DuoSet ELISA kit were used to quantify the extracellular concentration of those cytokines in the supernatant. Absorbance was determined using a spectrophotometer (Benchmark Microplate reader, Biorad, Cressier, Switzerland) at a wavelength of 450nm with the correction wavelength set at 570nm.

### Quantitative real-time PCR

U-87 MG cells were seeded in a six-well plate at a density of 1 × 10^6^ cells per well. After 24h of seeding, cells were treated with 20 ng/mL of free IL-12, ISN, and PLGA NPs. LPS was used as a positive control at 10 ng/mL. After 24h of exposure, total RNAs were isolated from the cells. Cell lysis was performed directly in the well, adding 100µL of BL + 1-thioglycerol (TG) buffer (Promega). The total RNA was extracted using ReliaPrep™ RNA Cell Miniprep System (Promega, Madison, WI, USA). Thermo Scientific^™^ NanoDrop^™^ 2000 Spectrophotometer (Agilent Technologies, Santa Clara, CA, USA) was used to analyze the RNA quality and calculate RNA concentration for cDNA. The reverse transcriptase reaction was performed using an Omniscipt RT system kit (Qiagen, Germany) according to the manufacturer’s protocol. Real-time PCR was performed using a 7500-fast real-time PCR system (Applied Biosystems). The threshold cycles (Ct) were calculated, and relative expression levels for each gene of interest were calculated using the Pfall method after normalization with glyceraldehyde-3-phosphate dehydrogenase (GAPDH) and tyrosine 3-monooxygenase/tryptophan 5-monooxygenase activation protein zeta (YWHAZ) as housekeeping genes [22]. Primers were purchased from Thermo Fisher Scientific (Zug, Switzerland). Details about the primers are included in Supplementary Information Table S1.

### Quantitative immunophenotyping analysis by flow cytometry

U-87 MG cells were seeded in a six-well plate at a density of 1 × 10^6^ cells per well and allowed to attach for 24h. The next day, cells were exposed to 20 ng/mL of free IL-12, ISN, and PLGA NPs. After 24 h and 48 h of treatment, cells were stimulated for 4 h with 2ug/mL of eBioscience™ Cell Stimulation Cocktail (ThermoFisher Scientific Inc., Zug, Switzerland) for intracellular p rotein staining. Cells were then trypsinized, washed, and fixed with eBioscience™ Intracellular Fixation & Permeabilization Buffer Set (ThermoFisher Scientific Inc., Zug, Switzerland). LIVE/DEAD™ Fixable Violet Dead Cell Stain was used to stain the dead cells (data not shown) and IL-12/IL-23 p40 Monoclonal Antibody, Phycoerythrin (PE) eBioscience™ (Invitrogen, Invitrogen, Thermo Fisher Scientific Inc., Zug, Switzerland) was used for the intracellular staining of IL-12. Data acquisition was performed on a BD LSR FORTESSA (BD Biosciences, San Jose, CA, USA), and flow cytometry data were analyzed using the FlowJo software (Figure S1 shows the used gating strategy).

### Statistical analysis

GraphPad Prism Software vs. 9.0 (GraphPad Software Inc.) was used to execute all statistical analyses. Differences between more than two related groups were analyzed using one-way analysis of variance (ANOVA). The one-way analysis of variance (ANOVA) was followed by a post-hoc Tukey test for multiple comparisons. Results are expressed as a mean ± standard deviation from a minimum of three independent experiments. Differences were considered significant at *p < 0.05, **p < 0.01, ***p < 0.001, ****p < 0.0001.

## Results

### Development and characterization of immunostimulatory nanoparticles

Our study aimed to develop ISN comprising either IL-12 loaded PLGA NPs, or IL-12 loaded PLGA/DOTAP NPs. The ultimate objective was to effectively deliver IL-12 to GBM cells and macrophages, inducing a shift in the tumor microenvironment by boosting the expression of pro-inflammatory cytokines. If successful, encapsulating IL-12 will prevent it from degradation post-administration, enabling a controlled release from the nanocarrier. PLGA, a biodegradable and biocompatible polymer, was selected because it permits controlled drug release over extended periods, ranging from weeks to months [23]. We hypothesized that the biological impact of IL-12 administered via ISN and CPN would occur through two mechanisms: (i) the IL-12 released from ISN interacting with IL-12 receptors on the surface of GBM cancer cells and macrophages, and (ii) the intracellular delivery of IL-12 facilitated by ISN.

PLGA NPs and ISN were successfully developed, and the hydrodynamic particle size, polydispersity index, and zeta potential were evaluated in different solvents (ultrapure water, NaCl 10 mM, PBS, and SIF) to better resemble the physiological conditions (Table 1). Across all solvents, the PLGA NPs presented an average hydrodynamic particle size ranging from 168 to 172 nm, with an average polydispersity index (PdI) between 0.01 and 0.11. Meanwhile, the ISN exhibited an average hydrodynamic particle size spanning from 165 to 183 nm and an average polydispersity index (PdI) between 0.01 and 0.06 (Table 1). In water, PLGA NPs showed a negative zeta potential of -24 ± 1 at pH 6 due to the negative charge of PLGA from deprotonated carboxylic end groups of lactic and glycolic acid [16]. The increase in zeta potential values across all buffers (PBS, NaCl 10 mM, and SIF) primarily occurs because of (i) significant dilution within the buffer solution and (ii) the binding of sodium counter-ions to the negatively charged PLGA [24, 25]. Since our goal was to create ISN to treat the aggressive brain cancer glioblastoma, both PLGA NPs and ISN were evaluated into SIF to better mimic the tumor microenvironment.

**Table 1:** Physicochemical properties of empty PLGA NPs and ISN in different solvents (ultrapure water, NaCl 10 mM, PBS, SIF)

Formulation	Solvent	Particle size (nm)	Polydispersity index	Zeta potential (mV)	Encapsulation efficacy (%)
PLGA NPs	Ultrapure water	172 ± 2	0.11 ± 0.02	− 24 ± 1	NA
	NaCl 10 mM	168 ± 7	0.00 ± 0.03	− 1 ± 2	NA
	PBS	171 ± 6	0.05 ± 0.05	− 1 ± 2	NA
	SIF	170 ± 7	0.01 ± 0.04	− 1 ± 2	NA
ISN	Ultrapure water	183 ± 6	0.05 ± 0.01	− 24 ± 1	99.9 ± 0.0
	NaCl 10 mM	165 ± 4	0.05 ± 0.07	− 4 ± 2	NA
	PBS	166 ± 5	0.06 ± 0.02	− 2 ± 1	NA
	SIF	168 ± 5	0.01 ± 0.03	− 1 ± 1	NA

Values are expressed as a mean ± standard deviation

*NA* not applicable, *PBS* phosphate-buffered solution, *SIF* simulated interstitial fluid

**Fig. 1:**
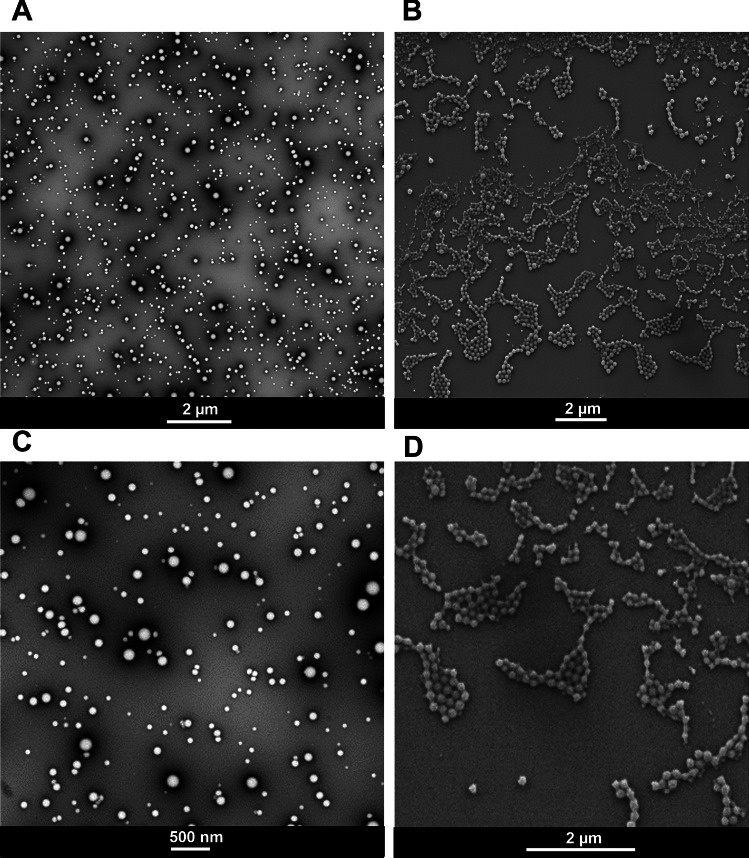
Morphology of immunostimulatory nanoparticles (ISN). The morphology of the nanoparticles was analyzed by **A**, **C** transmission electron microscopy (TEM) and **B**, **D** scanning electron microscopy (SEM). Scale bar of TEM microphotographs: 2 µm (**B**, **C**) and 500nm (**D**, **E**). Scale bar of SEM microphotographs: 2 µm

IL-12 is one of the most potent cytokines for anticancer immunotherapy with an effective concentration in a range between 0.2 and 100 ng/mL [26, 27]. For that reason, we could decrease the IL-12 drug loading percentage to low values (250 ng/formulation), resulting in high IL-12 encapsulation efficacy (around 99.9%) (Table 1). Regarding the morphology of the formulations, TEM and SEM microphotographs of ISN and PLGA NPs showed that all formulations had similar spherical shapes and smooth surfaces (Fig. 1 and S2).

### Development of hybrid cationic polymeric nanoparticles

**Fig. 2:**
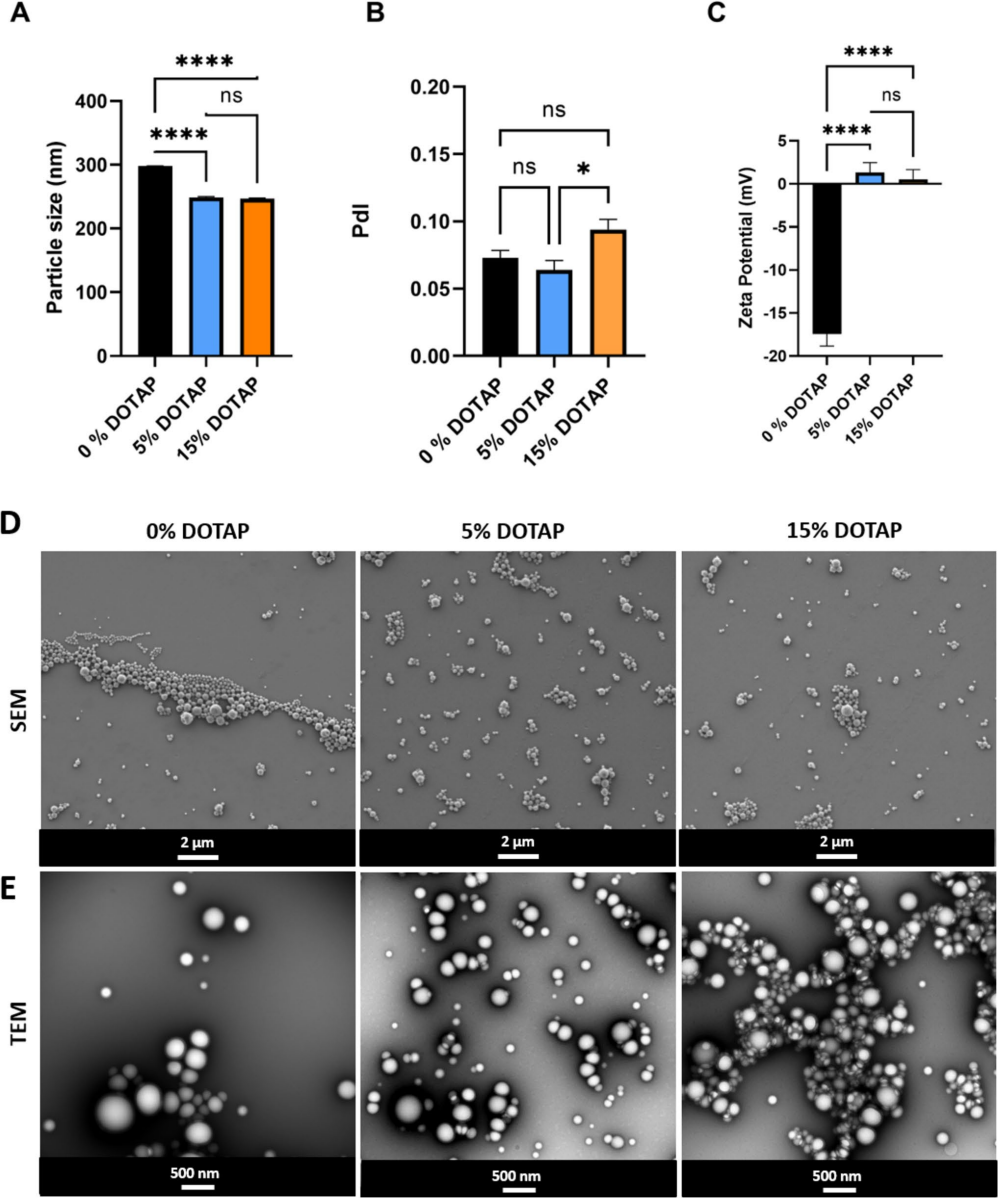
Development of empty cationic polymeric nanoparticles. Empty CPN were analyzed by **A** particle size, **B** polydispersity index (PdI), and **C** zeta potential. Different concentrations of DOTAP were incorporated into the PLGA matrix: 0, 5, and 15% (w/w). All data are presented as means ± SD. Graphs represent pooled data from at least three independent experiments. **D** Representative SEM microphotographs of CPN with 0, 5, and 15% DOTAP. Scale bar: 2 µm. Representative TEM microphotographs of empty CPN with 0, 5, and 15% of DOTAP. Scale bar: 500nm

To provide an efficient intracellular delivery of IL-12, we have developed CPN composed of PLGA and cationic lipid DOTAP. Three different ratios of DOTAP were incorporated into the PLGA matrix: 0, 5, and 15% (w/w). Figure 2A–C shows the physical–chemical characterization of empty CPN with varying percentages of DOTAP in the formulations whereas Fig. 2D and E shows the morphology of these respective formulations. Overall, empty CPN had an average hydrodynamic particle size of 220–300 nm (Fig. 2A), an average polydispersity index (PdI) between 0.05 and 0.12 (Fig. 2B) and showed a spherical morphology and smooth surface (Fig. 2D, E). Figure 2C demonstrates a notable increase in the zeta potential (-17 ± 1 to 1 ± 1), indicating the successful integration of DOTAP, a synthetic cationic lipid, into the PLGA matrix. However, there were no notable differences observed among the various levels of added DOTAP. Knowing the inherent toxicity of DOTAP, we have selected 5% of DOTAP in both empty and loaded CPN for the upcoming in vitro studies [28].

### Immunostimulatory nanoparticles have a better cell safety profile than cationic polymeric nanoparticles

**Fig. 3:**
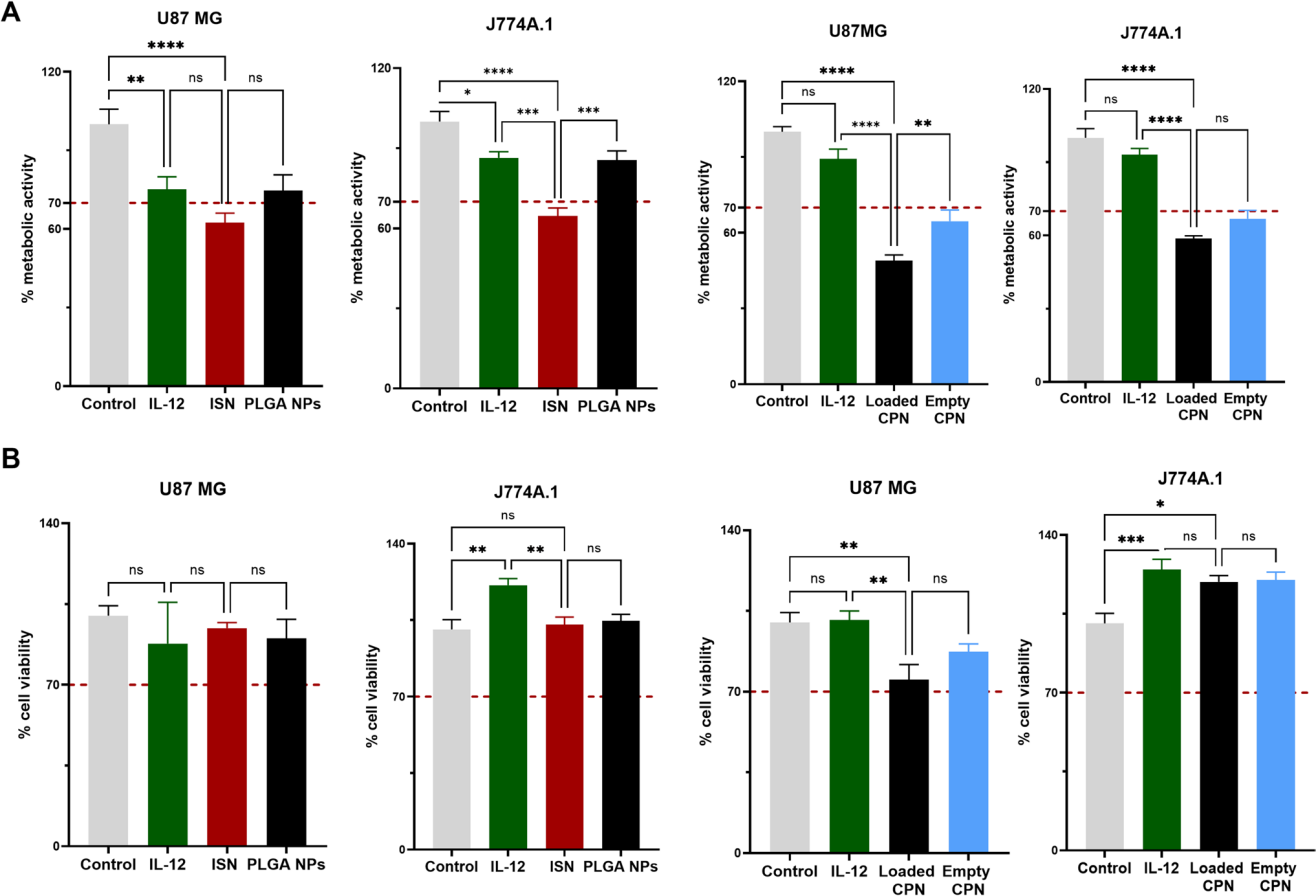
Immunostimulatory nanoparticles are less cytotoxic than cationic polymeric nanoparticles. **A** Percentage of metabolic activity measured by reduction of MTT after 24 h of exposure of free IL-12, ISN, and PLGA at 20 ng/mL in U-87 MG and J774A.1 cells. The metabolic activity of empty and loaded CPN was performed at 5 ng/mL. **B** Percentage of cell viability measured by LDH assay after 24 h of exposure of the previous treatment groups. The same concentrations were used in the LDH assay. In all graphs, bars represent mean values ± SEM (*n* = 8 values per condition with 3 biological experiments). Statistically significant differences among the groups (one-way ANOVA post-test for multiple comparisons). ns, non-significant, *p < 0.05, **p < 0.01, ***p < 0.001, ****p < 0.0001, determined as described in the “Materials and methods” section

Before the immunoactivity of ISN was studied, the cytotoxicity of all formulations was investigated. The metabolic activity of the cells was measured via the MTT reduction assay, while cell viability was determined by the LDH assay. Both GBM cells and J774A.1 macrophages were employed to investigate cell metabolic activity and cytotoxicity, aiming to eliminate formulations that exhibited significant toxicity compared to free IL-12. Figure 3A shows the metabolic activity of free IL-12, ISN, PLGA NPs, empty CPN, and loaded CPN, while Fig. 3B shows the cell viability profile of those formulations. Free IL-12 at 20 ng/mL, ISN, and PLGA NPs are not toxic for both cell lines. However, ISN reduced metabolic activity. On the other hand, both empty and loaded CPN at 5 ng/mL showed a significant decrease in metabolic activity (*p* < 0.0001), and they were toxic for the GBM cells. Given our primary objective to develop non-toxic nanoformulations for cytokine delivery as a cancer nanoadjuvant, both empty CPN and loaded CPN were excluded from the upcoming in vitro studies. Empty and loaded CPN were used at 5 ng/mL because at 20 ng/mL both formulations presented higher levels of toxicity (data not shown).

### Immunostimulatory nanoparticles stimulate the expression of pro-inflammatory cytokines

**Fig. 4:**
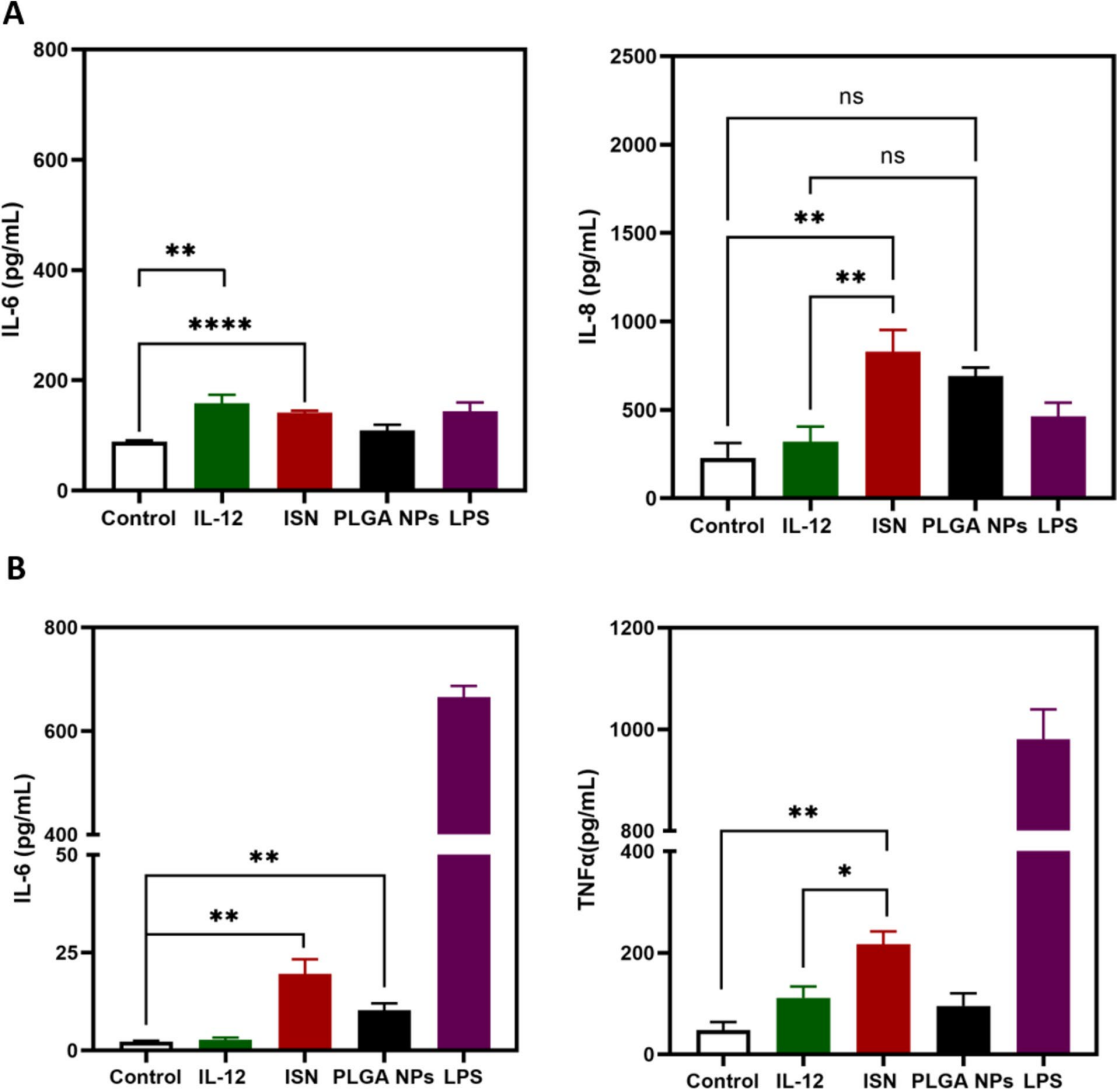
Immunostimulatory nanoparticles induced a higher release of pro-inflammatory cytokines. **A** Release of inflammatory cytokines in the cell culture media, interleukin-6 (IL-6) and interleukin-8 (IL-8), by U-87 MG cancer cells exposed to free IL-12, ISN, PLGA NPs at 20 ng/mL for 24h. Cells treated with lipopolysaccharide (LPS) were used as a positive control for inflammation. A supernatant was collected, and an enzyme-linked immunosorbent assay (ELISA) was performed as described in the "Materials and methods" section. **B** Release of inflammatory cytokines in the cell culture media, interleukin-6 (IL-6), and tumor necrosis factor-alpha (TNFα), by J77A.1 cells exposed to free IL-12, ISN, PLGA NPs for 24h. In all graphs, bars represent mean values ± SEM (*n* = 3 biological replicates). ns, non-significant, *p < 0.05, **p < 0.01, ****p < 0.0001, determined as described in the "Materials and methods"

After 24h of free IL-12, ISN and PLGA NPs exposure to GBM cells and J774A.1 macrophages, we evaluate the production of IL-6, IL-8, and TNF-α as pro-inflammatory cytokines (Fig. 4). IL-12 is a NF-kB-induced cytokine allowing the production of pro-inflammatory cytokines, such as IL-6, IL-8, and TNF-α [29]. For that reason, we compared the extracellular expression levels of those cytokines for the treatment’s groups: free IL-12, ISN, and PLGA NPs. After exposure to free IL-12, it was just observed a significant increase in the expression of extracellular IL-6 for the GBM cells (Fig. 4A). Surprisingly, ISN allowed a significant increase for both pro-inflammatory cytokines IL-6 and IL-8 in GBM cells and an increase for TNFα and IL-6 for J774A.1 macrophages. Empty PLGA NPs did not impact the expression of pro-inflammatory cytokines for the GBM cells. In contract to GBM cells, empty PLGA NPs impacted the expression of IL-6 in J774A.1 (Fig. 4B). It is well known that PLGA nanoparticles are highly uptaken by macrophages provoking distinct in vitro inflammatory responses [30]. Extracellular IFN-γ and VEGF levels were also measured with ELISA assay. However, no significant differences were found for all the groups (data not shown).

**Fig. 5:**
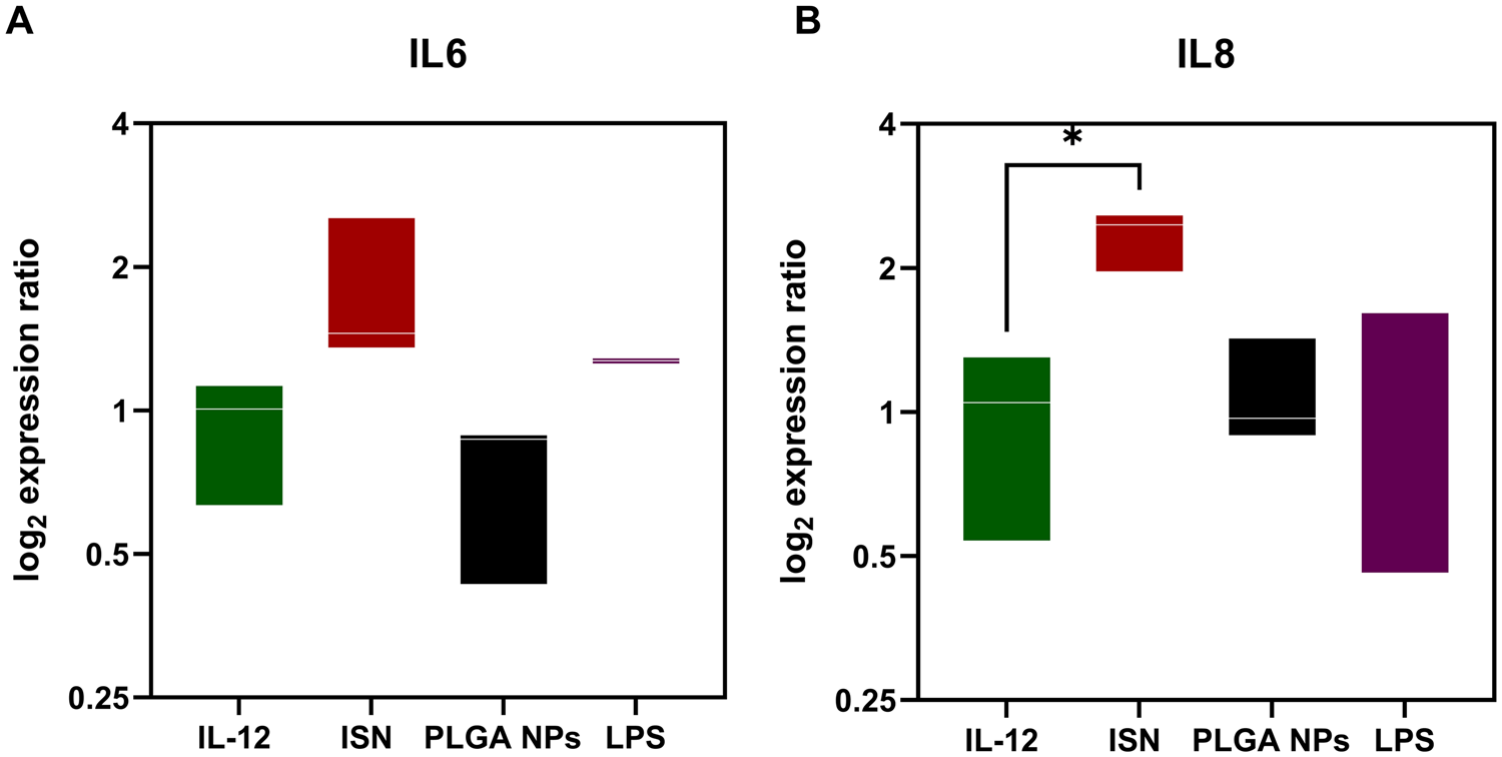
ISN induced an increase in gene expression for IL6 and IL8 genes upon exposure to U-87 MG cancer cells. **A** Real-time qRT-PCR results representing the expression of IL-6 and **B** IL-8 upon 24h of exposure to free IL-12, ISN, PLGA NPs, and LPS in human glioblastoma cancer cells (U-87 MG cell line). In all graphs, bars represent mean values ± SEM (*n* = 3 biological replicates). Statistically significant differences among the groups (one-way ANOVA post-test for multiple comparisons). *p < 0.05

To correlate the extracellular levels of pro-inflammatory cytokines with changes at the genetic level upon cell-nanoparticle uptake, real-time qRT-PCR was performed after 24h of nanoparticle exposure by GBM cells (Fig. 5). The results showed increased gene expression for IL-6 and IL-8 just for the ISN group. These results are in agreement with previous ELISA results, showing that ISN modulated the inflammatory response of GBM cells through several mechanisms that might be related to mitochondrial metabolism (as seen in Fig. 3A).

### Immunostimulatory nanoparticles allowed the intracellularly delivery of IL-12

**Fig. 6:**
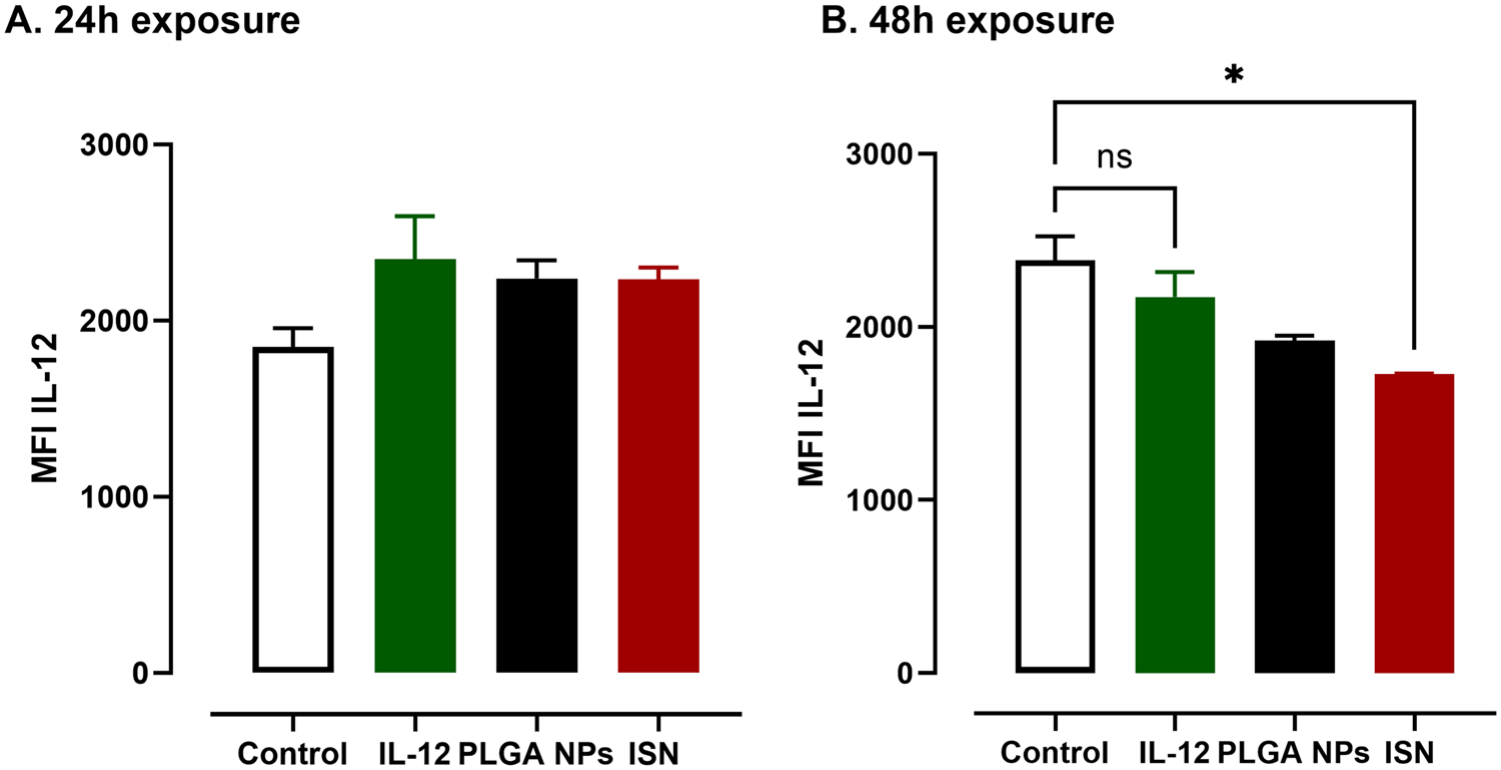
Immunostimulatory nanoparticles led to a decrease in intracellular IL-12. U-87 MG cancer cells were treated with medium (control), free IL-12, empty PLGA NPs, and ISN at a concentration of 20 ng/mL, and a quantitative immunophenotyping analysis by flow cytometry was performed after (**A**) 24h and (**B**) 48h of incubation. Bar graphs represent the median fluorescence intensity (MFI) of IL12 normalized to the control. The data are expressed as mean ± SD of three independent biological experiments. Statistically significant differences among the groups (one-way ANOVA post-test for multiple comparisons). ns, non-significant, *p < 0.05

It is well studied that PLGA nanoparticles can enable the intracellular delivery of drugs (including proteins and monoclonal antibodies) by GBM cells via endocytosis [31]. To better study this hypothesis, we have studied the detection of intracellular IL-12 after 24 h and 48 h by flow cytometry. Results revealed that after 48h of ISN exposure, GBM cancer cells could significantly decrease the intracellular amount of IL-12, suggesting that IL-12 was downregulated by ISN (Fig. 6). However, after 24h, no significant changes in the intracellular IL-12 content were observed for all the treatment groups.

## Discussion

IL-12 is a potent anticancer cytokine used as a combinatorial immunotherapy due to its pro-inflammatory properties. Within the tumor microenvironment, this cytokine can change cellular phenotypes from more inflamed to less inflamed, directly supporting the persistent cytotoxic activity of T cells and macrophages [32]. IL-12 is considered a powerful immunoadjuvant capable of trigger the immune response enhancing vaccine efficacy [33–35]. However, IL-12 is poorly tolerated when intravenously administered due to its high toxicity, short half-life, and serious adverse effects such as cytokine release syndrome and vascular leak syndrome [26]. To overcome those limitations, we have developed ISN composed of IL-12-loaded PLGA NPs to control the delivery, decrease the dose for further in vivo studies, decrease the toxicity, and allow a higher expression of pro-inflammatory cytokines achieved through (i) a controlled release of IL-12 and (ii) intracellular delivery. Figure 1 shows that ISN, with a spherical shape and a smooth surface, presented an average particle size of 183 ± 6 nm, a PDI of 0.05 ± 0.01, and a negative zeta potential of -24 ± 1. To improve the intracellular delivery of IL-12, we have developed CPN with different concentrations of the cationic lipid DOTAP. Since IL-12 was negatively charged at pH’s formulation, using a cationic lipid in the formulation would allow a strong ionic interaction between IL-12 and DOTAP. Figure 2 shows no significant differences between empty CPN with 5% of DOTAP and 15% concerning particle size, PdI, and zeta potential. Despite the several advantages of incorporating DOTAP in polymeric nanoformulations, it is well reported that high cell toxicity is associated with cationic lipid DOTAP [36]. Therefore, we have selected using 5% of DOTAP in both empty and loaded CPN for the in vitro cell viability studies with two selected cell types.

Metabolic activity and cell viability of GBM cells and J774A.1 macrophages were evaluated after exposure to free IL-12, ISN, PLGA NPs, empty CPN, and loaded CPN. Both empty CPN and loaded CPN showed a significant decrease in the metabolic activity for both cells (****p < 0.0001). We also observed a significant decrease in the cell viability for loaded CPN in GBM cells (Fig. 3B). The decrease in metabolic activity might be attributed to the toxicity of the cationic lipid DOTAP and the high concentration of PLGA and DOTAP [37]. This has been demonstrated by other researchers, wherein the toxicity of PLGA NPs escalated with higher concentrations of DOTAP [37]. Considering the inherent toxicity of DOTAP and observing favorable cell viability post-ISN exposure, the subsequent studies were conducted using the treatment groups: free IL-12, empty PLGA NPs, and ISN [38].

IL-12 is mainly synthesized by activated antigen-presenting cells such as macrophages, monocytes, dendritic cells, and B cells. IL-12 will stimulate pro-inflammatory cytokine expression and IFN-γ production, reprogramming CD4^+^ and CD8^+^ T cells to type 1 T helper (Th1) differentiation and reprogramming anti-inflammatory M2 phenotype macrophages to inflammatory M1 phenotype macrophages [5, 39]. To investigate the potential of ISN to induce an increase in pro-inflammatory cytokine expression, we have studied the release of two different pro-inflammatory cytokines from GBM cells and J774A.1 macrophages upon exposure. Figure 4A demonstrates a noteworthy increase in extracellular levels of IL-6 and IL-8 by GBM cells following exposure to ISN, contrasting with the response to free IL-12. In J774A.1 macrophages, a simultaneous and notable increase in both TNFα and IL-6 was observed specifically for the ISN group, demonstrating the ISN's capability to prompt higher expression levels of pro-inflammatory cytokines compared to free IL-12.

Since IL-12 production is regulated mainly at the transcriptional level, we compared gene expression with cytokine expression in GBM cells since our aim is to find a new treatment strategy for GBM [13]. It is well known that IL-12 leads to an increased expression of pro-inflammatory cytokines, however we also investigated the role of IL-6 and IL-8 for tumor immunosuppression in GBM. Figure 5 demonstrates an increase in the gene expression of both IL-6 and IL-8 in GBM cells, with the increase in IL-8 being statistically significant. These results were in agreement with ELISA results where it was showed also an increase of extracellular IL-8 for the ISN group (Fig. 3A). Our study showed that ISN loaded with IL-12 could modulate the inflammatory response of GBM cells at the transcriptional level, where IL-12 delivery upregulated other inflammatory genes. Apart from the immune system, stimulating GBM cancer cells to produce pro-inflammatory cytokines will activate the immune system to produce more pro-inflammatory cytokines and reprogram T cells and macrophages, inhibiting tumor growth. This study showed that ISN could empower glioblastoma immunotherapy compared with free IL-12. Similar studies have explored the intratumoral delivery of IL-12 mRNA therapy in other solid cancers, demonstrating its ability to facilitate a transformation in the tumor microenvironment and provoke robust antitumor immunity [40, 41]. Focusing on GBM, the advancement of ISN as a cancer nanoadjuvant marks a novel area that could potentially unlock the development of a new form of immunotherapy. However, conducting in vivo studies encompassing pharmacokinetic and pharmacodynamic profiles of ISN would be advisable as a pre-clinical model for evaluating a new glioblastoma treatment.

Finally, one of our objectives was to investigate the intracellular delivery of cytokines through PLGA NPs. We demonstrated that ISN could effectively modulate intracellular levels of pro-inflammatory cytokines, facilitating a significant enhancement in inflammation. Figure 6 shows a decrease in the intracellular levels of IL-12 upon 48 h of ISN exposure to GBM cells underlying downregulation of IL-12 secretion. We might conclude that ISN allowed an intracellular delivery providing a downregulation over time for the IL-12 expression. Even though the molecular mechanism approach was not evaluated in this study, this study opens doors for researchers to address how released IL-12 might interfere with the IL-12 production at transcriptional level. Wang et al. also showed the same trend where authors have shown that an intracellular accumulation of IL-12 by oncolytic adenovirus may act to downregulate the IL-12 expression at transcriptional level once an intracellular threshold was reached [26]. However, the molecular mechanism behind the downregulation is not yet understood.

To our knowledge, this is the initial study reporting the successful delivery of IL-12 to GBM cells using biocompatible and biodegradable nanoparticles. IL-12, a potent anticancer cytokine employed in cancer immunotherapy, had its use discontinued due to the various toxicities observed in clinical trials. Our study proved that the encapsulation of IL-12 allowed a decrease in toxicity and promoted the expression of pro-inflammatory cytokines by J774.1 macrophages and GBM cells. In fact, the expression of pro-inflammatory cytokines was higher for the ISN compared to the free IL-12 (Figs. 4 and 5). The therapeutic efficacy of ISN loaded with IL-12 underlies its ability to deliver extracellularly and intracellularly IL-12 and trigger a potential downregulation mechanism reducing the intracellular levels of IL-12. Delivering cytokines to the local site via ISN opens doors for deep fundamental research regarding the molecular mechanisms underlying GBM cytokine dynamics.

## Conclusions

In our research, we have pioneered the development of ISN loaded with the potent cancer cytokine IL-12, aiming to boost the immune response against GBM and establish it as a cancer nanoadjuvant for GBM vaccination. Our findings strongly indicate that ISN holds promise as a platform to manipulate the glioblastoma microenvironment by intensifying inflammation, thereby bolstering glioblastoma immunotherapy. Leveraging nanocarriers, our created cancer nanoadjuvant can potentially co-encapsulate other immune-activating cytokines and be utilized in combination therapies with chemotherapy or other immunotherapies. However, further in vivo investigations are crucial to replicate the tumor immune microenvironment and determine the optimal administration route for ISN to penetrate the brain. Despite the excellent safety profile of PLGA NPs in both in vivo and patient contexts, our nanosystem must undergo rigorous safety assessments concerning the delivery of a potent pro-inflammatory cytokine like IL-12. Prolonged delivery of IL-12 could potentially trigger localized inflammation in the body, potentially leading to neuroinflammation. Irrespective of these studies, our data clearly shows effectiveness in enhancing inflammation through IL-12 delivery.

## Supplementary Information

Below is the link to the electronic supplementary material.

Supplementary file1 (DOCX 3125 KB)

## Data Availability

The data generated during and/or analyzed during the current study are available from the corresponding author on reasonable request.
