# Supplementary material for: Immunostimulatory nanoparticles delivering cytokines as a novel cancer nanoadjuvant to empower glioblastoma immunotherapy
Source: Drug Deliv Transl Res. 2023 Dec 31;14(10):2655–67. doi: 10.1007/s13346-023-01509-2 (PMC11385014; doi:10.1007/s13346-023-01509-2)
Supplement: Supplementary file 1 — Supplementary file1 (DOCX 3125 KB) [file 13346_2023_1509_MOESM1_ESM.docx]

Supplementary Material

**Immunostimulatory nanoparticles delivering cytokines as a novel cancer nanoadjuvant to empower glioblastoma immunotherapy**

Flávia Sousa^1,2*^, Henry Lee^1^, Mauro Almeida^1^, Amelie Bazzoni^1^, Barbara Rother-Rutishauser^1,2^, Alke Petri-Fink^1,3^

1 Adolphe Merkle Institute, University of Fribourg, Switzerland

2 National Center of Competence in Research Bio-Inspired Materials, University of Fribourg, Switzerland

3 Chemistry Department, University of Fribourg, Chemin du Musée 9, 1700 Fribourg, Switzerland

*** Correspondence:**

Flávia Sousa [flavia.sousa@unifr.ch](mailto:flavia.sousa@unifr.ch)

Adolphe Merkle Institute, University of Fribourg, Switzerland


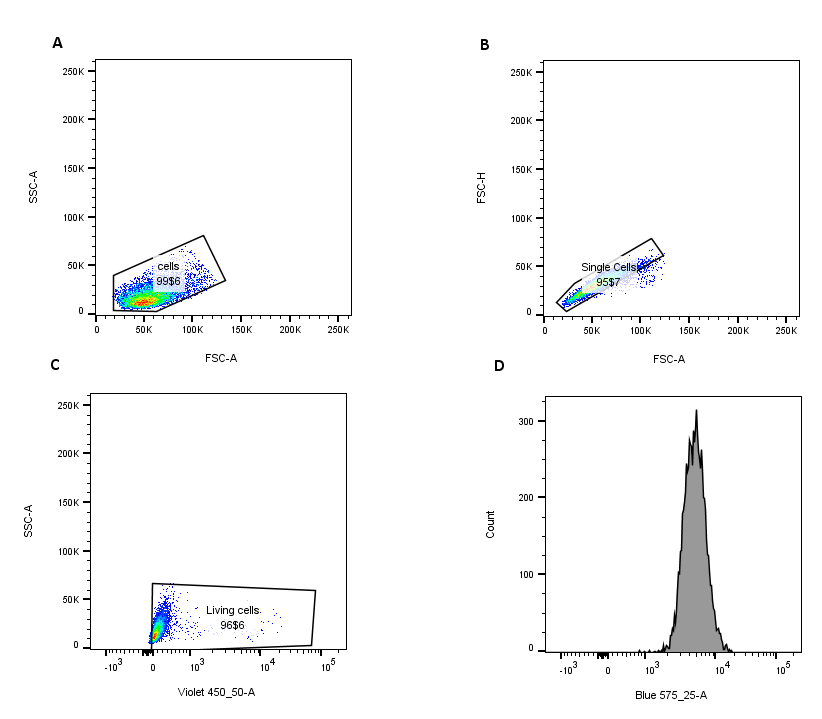


**Figure S1**. **Gating strategy for flow cytometry.** (A) At first, U87-MG cells were gated using forward and side scatter area (FSC-A and SSC-A, respectively) to identify the cells of interest**.** (B) Next, to remove the doublets, a new gate was performed using forward scatter height and forward scatter area (FSC-H and FSC-A, respectively). (C) To remove dead cells, LIVE/DEAD™ Fixable Violet Dead Cell Stain was used, a new gate was used for side scatter area and Fixable Violet Dead Cell. (D) Single-parameter histograms were used to identify intracellular IL-12.


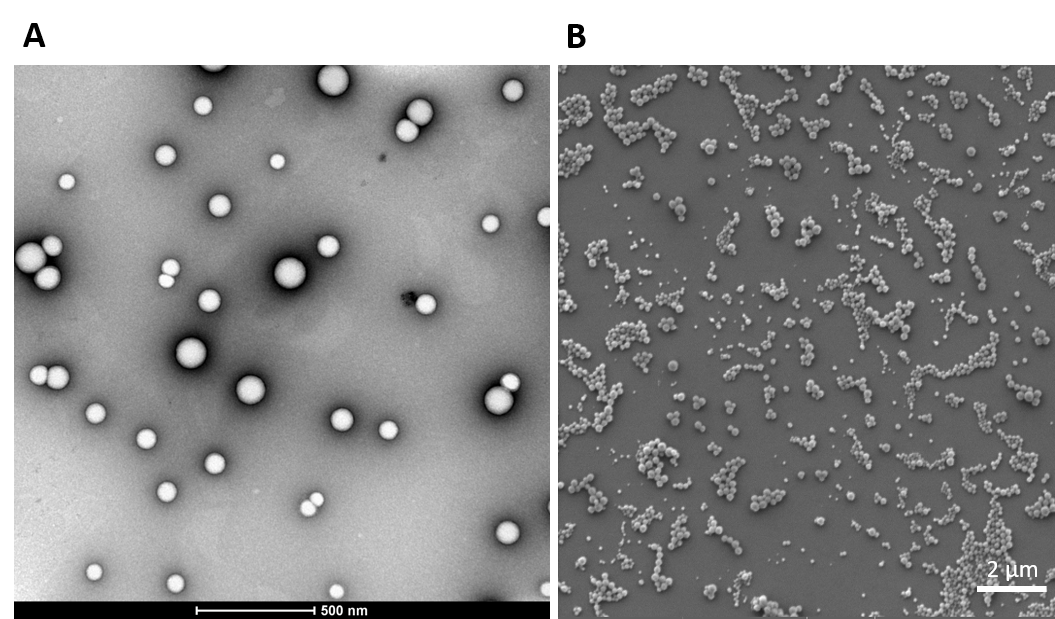


**Figure S2**. Morphology of Empty PLGA NP. The morphology of the Empty PLGA NP was analyzed by (E) transmission electron microscopy (TEM) and (F) scanning electron microscopy (SEM). Scale bar of TEM microphotographs: 500nm. Scale bar of SEM microphotographs: 2 µm.

**Table S1**. Information about the primers used for Real-time qRT-PCR. FW: Forward. RV: Reverse.

| Primers | Gene symbol | Gene name | Sequence (5’ – 3’) | Product length | Efficiency |
| --- | --- | --- | --- | --- | --- |
| IL-6 | IL6 | Interleukin 6 | FW:CCACTCACCTCTTCAGAACG | 25 | 93% |
|  |  |  | RV:CATGTTACTCTTGTTACATGTCTCC |  |  |
| IL-8 | CXCL-8 | C-X-C motif chemokine ligand 8 | FW: GAGAAGTTTTTGAAGAGGGCTGA | 91 | 90% |
|  |  |  | RV: GCTTGAAGTTTCACTGGCATCT |  |  |
| Housekeeping | GAPDH | Glyceraldehyde-3- phosphate dehydrogenase | FW: GTCGGAGTCAACGGATTTGG | 147 | 82% |
|  |  |  | RV: GCCATGGGTGGAATCATATTGG |  |  |
|  | YWHAZ | Tyrosine 3- monooxygenase/tryptoph an 5-monooxygenase activation protein zeta | FW: GCTGGTGATGACAAGAAAGGGAT | 120 | 90% |
|  |  |  | RV: GTTAAGGGCCAGACCCAGTC |  |  |
